# No small dilemma: small bowel volvulus mimicking acute coronary syndrome

**DOI:** 10.1093/omcr/omae080

**Published:** 2024-07-30

**Authors:** Cristian D Armas, Sean Bademian, Mikaela Kcomt, Jessica Burgess, Xian Qiao

**Affiliations:** School of Medicine, National University of Trujillo, Av. Roma 338, Trujillo 13011, Peru; Department of Internal Medicine, Division of Pulmonary Disease and Critical Care, Eastern Virginia Medical School, 825 Fairfax Ave, Norfolk, VA 23507, United States; School of Medicine, Universidad Privada Antenor Orrego, Av. America Sur 3145, Trujillo 13008, Peru; Department of Surgery, Eastern Virginia Medical School, 825 Fairfax Ave, Norfolk, VA 23507, United States; Department of Internal Medicine, Division of Pulmonary Disease and Critical Care, Eastern Virginia Medical School, 825 Fairfax Ave, Norfolk, VA 23507, United States; Pulmonary, Critical Care, and Sleep Specialists, Sentara Medical Group, 600 Gresham Dr, Norfolk VA 23507, United States

**Keywords:** Wellens syndrome, volvulus, mesenteric ischemia, abdominal pain, bowel necrosis, acute coronary syndrome

## Abstract

Acute abdominal pathologies can cause electrocardiogram (ECG) changes mimicking an acute coronary syndrome (ACS), resulting in diagnostic uncertainty and delay. We report a 65-year-old male with multiple risk factors for ACS who presented with four hours of progressive epigastric and chest pain that resolved in the emergency department. ECG findings were concerning for new deeply inverted T-waves with normal troponins, raising concerns for Wellens Syndrome. Emergent heart catheterization was negative but abdominal computed tomography angiography showed occlusion of the superior mesenteric vessels. Subsequent exploratory laparotomy revealed a small bowel volvulus with extensive necrosis, resulting in a 430 cm resection.

## Introduction

ST-elevation myocardial infarctions (STEMIs) are medical emergencies and a door-to-balloon time of 90 min or less is recommended as best practice [1, 2]. Deeply inverted or biphasic T-waves in the precordial leads with minimal or no ST elevations raise concerns for Wellens Syndrome, which is a well-documented STEMI-equivalent finding that typically involves stenosis of the proximal portion of the left anterior descending (LAD) coronary artery [3]. However, dynamic ECG changes, like those seen in Wellens Syndrome, can also occur in acute abdominal pathologies such as mesenteric ischemia, appendicitis, bowel obstruction, pancreatitis, and cholecystitis (Table 1). Here, we present the first reported case of small bowel volvulus with necrosis presenting with an ischemic pattern on ECG concerning for Wellens Syndrome and no significant ischemia on coronary angiography, along with a review of the available case reports for abdominal pathology induced ischemic changes on ECG.

**Table 1:** Summary of acute abdominal pathology cases presenting with transient ischemic ECG patterns, resembling acute coronary syndromes

Abdominal pathology	Author/Year	Age/Gender	Symptoms	ECG changes	Cardiac tests	Cardiac enzymes (ng/ml)	Outcomes
Small bowel necrosis	Koci F, et al. 2014	63 F	Abdominal pain, diaphoresis, nausea, chest pain	Borderline (<1 mm) STE in V2 and V3 Significant loss of R waves in precordial leads	Catheterization: No significant coronary lesions. TTE: Balloon-like configuration of severely hypokinetic mid-distal left ventricular wall with preserved contractility of the basal portion.	Troponin T: 0.13 (negative: <0.03)	Discharged after small bowel resection (5-day ICU stay). Developed Takotsubo cardiomyopathy; follow-up TTEs during the next 2 years revealed normalization of left ventricular function.
Acute mesenteric ischemia	Yeh YT, et al. 2015	61 M	Epigastric pain, vomiting, watery diarrhea	Q waves with STE in inferior leads	Catheterization: 70% stenosis in the first diagonal branch of the LAD coronary artery.	Troponin T: 0.028 (negative: <0.014)	Discharged after right hemicolectomy (10-day hospitalization). ECG changes resolved after surgery.
Acute mesenteric ischemia	Baldeo C, et al. 2018	70 F	Epigastric pain, chest pain, dyspnea	Atrial fibrillation with new STE in inferior leads	Catheterization: Native coronary artery disease with patent grafts and no evidence of new obstructive disease. TTE: Left ventricular apical ballooning and hyperkinetic base, LVEF of 40-45%.	Troponin T: 0.28 (negative: <0.04)	Discharged after caecum resection. ECG changes resolved after surgery. Developed Takotsubo cardiomyopathy; TTE prior to discharge showed improved left ventricular function with LVEF of 45-50%.
Acute appendicitis	Dewar C, et al. 2002	54 M	Central abdominal pain, nausea	STE in anteroseptal leads	N/A	Not elevated	Discharged a few days after appendectomy (9-day hospitalization); complete recovery. ECG changes resolved after surgery.
Gastric ulcer perforation	Intan RE, et al. 2021	70 M	Severe epigastric pain, nausea and vomiting, constipation, fever, lethargy	STE in anteroseptal leads (1 mm in V1, 2 mm in V2 and V3)	TTE: Normal.	CK-MB: Not elevated Troponins not evaluated	Discharged 4 days after exploratory laparotomy without any remaining symptoms. ECG changes resolved after surgery.
Gastric ulcer perforation	Vutthikraivit W 2020	78 M	Severe epigastric pain	STE in V2 and V3	TTE: Normal. Coronary CTA: No significant stenosis of coronary arteries.	Troponin T: <0.01	Discharged 7 days after exploratory laparotomy and simple suture of perforated gastric ulcer; no complications during the postoperative course. ECG changes resolved after surgery.
Distended stomach conduit	Asada S, et al. 2005	83 F	Chest discomfort	STE in II, III and aVF Prominent negative P wave in V1	Catheterization: Normal coronary arteries. TTE: Left atrial and ventricular compression by a distended stomach conduit.	Not elevated	ECG changes resolved after nasogastric decompression.
Colonic ileus	Zhang, et al. 2020	41 M	Abdominal distention, shortness of breath, fatigue, lower extremity edema	Sinus tachycardia STE with deep T-wave inversions in precordial leads Marked QT prolongation	TTE: Normal.	Troponin T: <0.01	ECG changes resolved after nasogastric decompression. Patient died during ICU hospitalization due to metastatic lymphoma complicated by septic shock, acute respiratory failure, and acute kidney injury.
Small bowel ileus	Hibbs, et al. 2016	34 F	Abdominal pain, nausea, emesis	Sinus tachycardia Prominent J waves STE in II, III, aVF, V4 to V6	TTE: Hyperdynamic heart with normal LVEF. Coronary CTA: Normal coronary arteries.	Troponin I < 0.01	Managed conservatively with intravenous rehydration and antiemetic. Complete recovery. ECG changes resolved after treatment.
Acute cholecystitis	D’Alessandro A, et al. 2019	75 M	Fever	STE in II, III, aVF, V5 and V6 Nonspecific T-wave inversion in III Hyperacute T-waves in anterior leads	TTE: Normal.	Troponins: Not elevated	N/A
Colonic obstruction	Herath H, et al. 2016	56 M	Abdominal discomfort and fullness, nausea and vomiting, sweating	STE with T-wave inversion in V1 to V3	TTE: Normal.	Troponin I: <0.10 CK-MB: 3.34 Myoglobin: 29.94	Discharged after nasogastric decompression (5-day hospitalization); complete recovery. ECG changes resolved after nasogastric decompression.
Partial small bowel obstruction	Mixon, et al. 2003	64 F	Abdominal pain, nausea, emesis, fever	STE in V1 to V3	TTE: Right atrial and ventricular compression by a distended jejunal conduit.	Not elevated	ECG changes resolved after nasogastric decompression.
Small bowel obstruction	Patel, et al. 2015	42 F	Epigastric discomfort, nausea and vomiting, shortness of breath	STE in lateral and inferior leads	Catheterization: Normal coronary arteries. TTE: Inferior wall hypokinesis.	Not elevated	ECG changes resolved after exploratory laparotomy with small bowel resection and anastomosis.
Small bowel obstruction	Parikh, et al. 2015	86 M	Epigastric pain, nausea and vomiting	STE in inferior leads with reciprocal changes in anterior precordial leads	Catheterization: 70% stenosis of the right coronary artery, 40% stenosis of the LAD coronary artery, none of the vessels showed plaque rupture or acute thrombus.	Troponin T: 0.04	Complete recovery. ECG changes resolved after nasogastric decompression.
Small bowel obstruction	Upadhyay, et al. 2017	64 M	Abdominal pain, constipation, nausea and vomiting	Sinus tachycardia STE in inferior and lateral leads	TTE: Normal	Troponin T: 0.048 CK-MB: Not elevated	ECG changes resolved after exploratory laparotomy.
		71 F	Epigastric pain, constipation	STE in II, III, aVF, V5, and V6	Catheterization: Normal coronary arteries, LVEF of 35%, diaphragmatic and posterobasal hypokinesis on ventriculogram.	Troponins up to 5	Discharged a few days after exploratory laparotomy with small bowel resection and anastomosis. ECG changes resolved after surgery. Follow-up TTE: Normal.
Acute pancreatitis	Pezzilli, et al. 2010	36 M	Severe epigastric pain	STE of 1 mm in II, III, V2 and V3 Inverted T-waves in V4 to V6 Isolated Q wave in III	Catheterization: Normal coronary arteries. TTE: Normal.	Troponin I: <0.04 (normal: 0.04–0.14)	Received supportive care (intravenous fluids, analgesia) and recovered in 6 days. ECG changes resolved gradually towards his baseline.
Acute pancreatitis	Agrawal, et al. 2018	60 M	Epigastric pain, syncope	Sinus bradycardia Peaked T-waves and STE of 1 mm in II, III, and aVF STE of 2 mm in V3	Catheterization: Nonobstructive coronary artery disease. TTE: Normal.	Troponins: <0.01	Received aggressive fluid resuscitation. Recovered in 3 days. ECG at the time of discharge showed persistent (baseline) STE in V2 and V3.
Acute pancreatitis	Antonelli, et al. 2017	46 M	Mild upper abdominal pain radiating to the back, nausea, chest discomfort radiating to the neck	Large peaked T-waves in II, III, and aVF Giant T-wave inversion in aVL, V1 to V6	Catheterization: Normal coronary arteries. TTE: Normal.	Troponin T: <0.01	Managed conservatively. Complete recovery. ECG changes and chest discomfort resolved 30 min after admission.

STE = ST-segment elevation; TTE: transthoracic echocardiogram; CTA = computed tomography angiography; N/A = not applicable; LVEF = left ventricular ejection fraction.

## Case report

**Figure 1:**
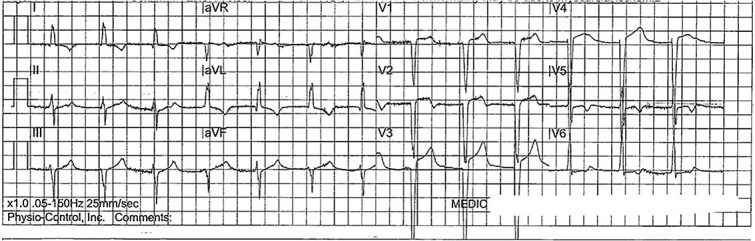
ECG taken en route to the Emergency Department showing ST elevation in the anterior-lateral leads.

**Figure 2:**
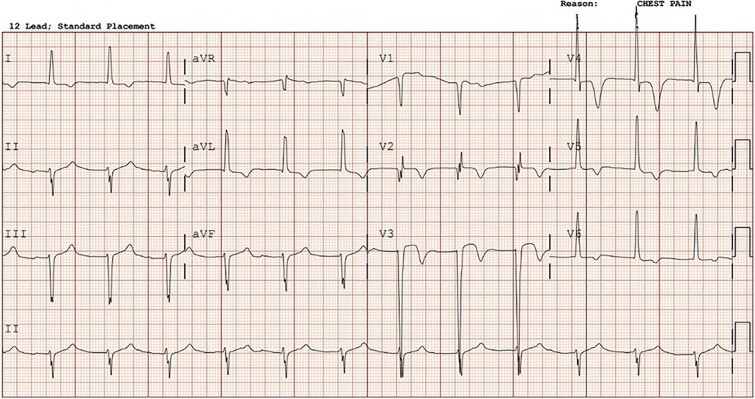
ECG in the Emergency Department revealing an accelerated junctional rhythm with minimal ST elevation in V3, and new deeply inverted T waves in V2-V4 consistent with Wellen’s T’s, concerning for ischemic changes.

**Figure 3:**
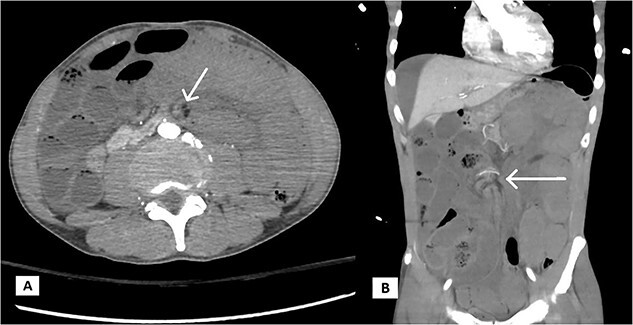
Computed tomography angiography of the abdomen with areas of “swirls” (white arrows) concerning for volvulus. A, axial and B, coronal views.

A 65-year-old male active smoker with a history of non-ischemic cardiomyopathy, hypertension, and hyperlipidemia was brought to the hospital by ambulance after four hours of progressive epigastric and chest pain. During transit, ECG showed ST elevation in the anterior-lateral leads (Fig. 1). Repeat ECG In the emergency department revealed an accelerated junctional rhythm with minimal ST elevation in V3, lack of Q-waves, and new deeply inverted T waves in V2-V4 (Fig. 2). Physical examination showed stable hemodynamics, a distressed appearance with diaphoresis, and epigastric tenderness with guarding. Initial lab results showed an undetectable troponin level but elevated serum lactate of 5.8 mmol/l. The patient was emergently taken for left heart catheterization for concern of acute coronary syndrome (ACS). However, the catheterization showed no significant coronary disease and normal filling pressures. Post-procedurally, the patient continued to experience severe abdominal pain. A computed tomography angiography (CTA) of the abdomen suggested occlusion of the superior mesenteric artery and vein with extensive small bowel ischemia (Fig. 3). During the exploratory laparotomy, the vascular team discovered a small bowel volvulus with necrotic bowel extending from the mid-jejunum to the terminal ileum, requiring an extensive 430 cm small bowel resection by general surgery. Post-laparotomy ECG showed resolution of all previously noted changes and normalization of lactic acid levels (1.9 mmol/l).

## Discussion

Wellens Syndrome on ECG consists of deeply inverted or biphasic T-waves in multiple precordial leads, coupled with an isoelectric or minimally elevated ST-segment (<1 mm), lack of Q-waves, and preservation of normal R-wave progression [3, 4]. This ECG pattern was first identified by de Zwaan et al in 18% of patients admitted for unstable angina [3]. Among these patients, 75% who did not undergo coronary revascularization eventually experienced a severe anterior wall infarction within a few weeks [3]. Subsequently, a study by the same researchers confirmed a 100% association with significant proximal LAD artery blockage, ranging from 50% to complete obstruction [5]. As a result of these findings, Wellens syndrome is considered a “STEMI-equivalent,” despite cardiac enzyme levels being usually normal or only slightly elevated [3, 4]. Unfortunately, our patient lacked the normal R-wave progression due to left ventricular hypertrophy, thereby cannot be considered to have true Wellens Syndrome. Nonetheless, new T-wave inversions >2 mm in anterior leads as a sole indicator have a high positive predictive value (86%), sensitivity (69%), and specificity (89%) for significant LAD disease [6]. Definitive management in either case involves urgent coronary angiography to assess the severity of coronary artery blockage [4].

However, ECG changes are not solely related to cardiac pathology. In the existing literature, several case reports describe transient ischemic ECG patterns associated with acute abdominal pathologies resembling ACS. This includes three cases of mesenteric ischemia presenting as ST elevation on ECG. The first case involved a novel presentation of mesenteric ischemia mimicking inferior STEMI in a 61-year-old male who presented with epigastric pain, vomiting, diarrhea, and an increase in troponin levels. ECG changes resolved following right hemicolectomy [7]. Another case represents a rare presentation of small bowel strangulation due to adhesions, which caused bowel infarction in a 63-year-old female who exhibited borderline anterior ST elevation and developed Takotsubo cardiomyopathy secondary to physical stress [8]. The last case involved stress-induced cardiomyopathy in a 70-year-old female who presented with acute-onset chest pain, inferior ST elevation, rising troponin levels, and elevated lactate, leading to caecum resection and subsequent ECG normalization [9]. T-wave inversions and ST changes can also be observed in acute pancreatitis, acute cholecystitis, gastric distention, and bowel obstruction. Table 1 summarizes some remarkable cases resembling ACS.

The underlying pathophysiology of dynamic ECG changes seen with acute abdominal pathologies remains unclear and has not been fully investigated or published. It is likely a multifactorial process and several mechanisms have been proposed. The first mechanism involves the distension of a hollow organ, which can directly compress or displace the heart within the thoracic cavity [10]. Similarly, increased intra-abdominal pressure can result in the relative displacement or compression of the inferior surface of the heart, leading to changes in the QRS axis and/or voltage. A second proposed mechanism is based on the occurrence of a vasovagal reflex due to gastrointestinal tract distention. This visceral-cardiac reflex causes increased vagal tone, potentially causing ECG changes and transient coronary vasospasm. Finally, a similar mechanism to Takotsubo cardiomyopathy has been proposed, where an exaggerated sympathetic stimulation and elevated plasma catecholamines due to emotional or physical stress results in microvascular spasm or dysfunction with myocardial stunning [11, 12].

Our case was particularly challenging because the patient presented with symptoms consistent with ACS, in addition to an abnormal abdominal exam and gastrointestinal complaints. Given the presence of significant coronary risk factors, STEMI-equivalent ECG changes, and timing recommended by the societal guidelines, cardiac evaluation took precedence. Unfortunately, this prioritization resulted in a delay in diagnosing small bowel volvulus with mesenteric ischemia, which triggered one or more of the pathophysiological mechanisms described above, resulting in Wellens T’s on ECG concerning for ischemia.

In summary, associating ischemic patterns on ECG with ACS is crucial, but it may be equally important to consider abdominal ischemia. A delay in diagnosing bowel ischemia contributes to the extent of tissue ischemia and resultant necrosis, as it similarly does to the heart and other organs. To the best of our knowledge, this is the first reported case of small bowel volvulus induced mesenteric ischemia presenting with ischemic patterns on ECG. We believe it highlights the unique association between ischemic changes on ECG and abdominal pathology, which can present the physician with a life-threatening diagnostic dilemma when timely cardiac or abdominal reperfusion is crucial.
